# Supplementary material for: Identification of photoperiod-induced specific miRNAs in the adrenal glands of Sunite sheep (Ovis aries)
Source: Front Vet Sci. 2022 Jul 22;9:888207. doi: 10.3389/fvets.2022.888207 (PMC9354845; doi:10.3389/fvets.2022.888207)
Supplement: Supplementary file 1 [file Data_Sheet_1.ZIP › Supplementary file/miRDeep2 file.zip!/miRDeep2 file/Novel_Structure/SP42A2/Novel_151.pdf]

Provisional ID	:	NC_019458.2_1689
Score total	:	2.5
Score for star read(s)	:	-1.3
Score for read counts	:	0
Score for mfe	:	2.2
Score for randfold	:	1.6
Score for cons. seed	:	
Total read count	:	208
Mature read count	:	208
Loop read count	:	0
Star read count	:	0

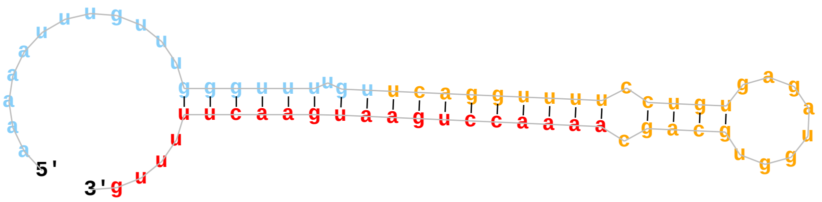

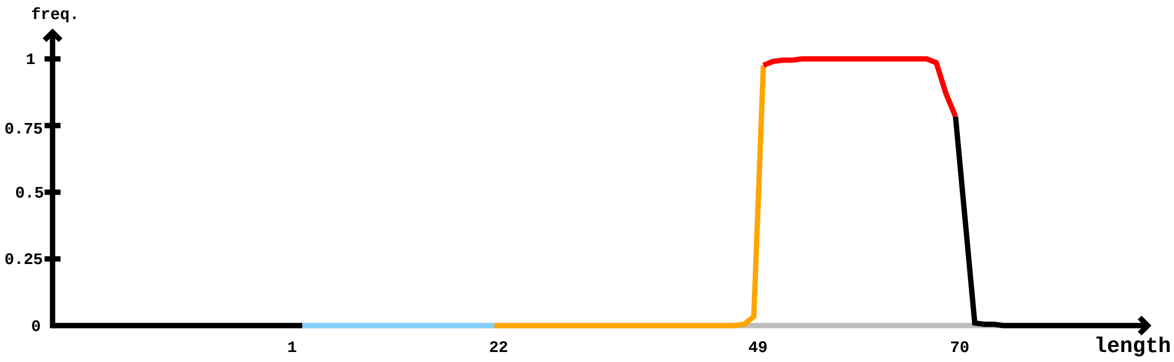


```
                                   Star                                             Mature
5'- uuaucagaaguauuggguuggccaaaaaauuuguuuggguuuuguucagguuuuccugugagauggugcagcaaaaccugaaugaacuuuuuggccagcccaauguuauuug   -3'    exp
    ....((((((((((((((((((((((((.(((((((((((((((((((...((((....))))...)).))))))))))))))))).)))))))))))))))))))).))))    reads     mm     sample
    ......................................................................gcaaaaccugaaugaacuuuuu....................    1         0      seq
    .......................................................................Uaaaaccugaaugaacuuu......................    1         1      seq
    .......................................................................Uaaaaccugaaugaacuuuuu....................    1         1      seq
    .......................................................................caaaaccugaaugaacuuuuu....................    2         0      seq
    .......................................................................Gaaaaccugaaugaacuuuuug...................    1         1      seq
    .......................................................................Uaaaaccugaaugaacuuuuug...................    1         1      seq
    ........................................................................aaaaccAgaaugaacuuu......................    2         1      seq
    ........................................................................aaaaUcugaaugaacuuuu.....................    23        1      seq
    ........................................................................aaaaccugaaugaacCuuu.....................    1         1      seq
    ........................................................................aaaaccAgaaugaacuuuuu....................    9         1      seq
    ........................................................................Gaaaccugaaugaacuuuuu....................    2         1      seq
    ........................................................................aaaaccugaaugaacuuuuC....................    1         1      seq
    ........................................................................aaaCccugaaugaacuuuuu....................    1         1      seq
    ........................................................................aaaaUcugaaugaacuuuuug...................    57        1      seq
    ........................................................................aaaaccugaauAaacuuuuug...................    1         1      seq
    ........................................................................aaaaccAgaaugaacuuuuug...................    2         1      seq
    ........................................................................aaaaccugaaugaacuuuuug...................    18        0      seq
    ........................................................................aaaaccugaaGgaacuuuuug...................    1         1      seq
    ........................................................................aaaaccugaaugaacuuCuugg..................    1         1      seq
    ........................................................................aaaaccugaaugaacuuuuugg..................    56        0      seq
    ........................................................................Gaaaccugaaugaacuuuuugg..................    3         1      seq
    ........................................................................aaaaccugaaugaacCuuuugg..................    1         1      seq
    ........................................................................aaaaccugaaugaacuuuuugA..................    16        1      seq
    ........................................................................aaaaccugaaugaacuuuuuggG.................    1         1      seq
    .........................................................................aaaccugaaugaacuuuuu....................    1         0      seq
    .........................................................................aaaccugaaugaacuuuuugA..................    2         1      seq
    ..........................................................................aaccugaaugaaAuuuuug...................    1         1      seq
    ............................................................................ccugaaugaacuuuuuggcca...............    1         0      seq
```
